# Gene expression of pro-inflammatory (IL-8, IL-18, TNF-α, and IFN-γ) and anti-inflammatory (IL-10) cytokines in the duodenum of broiler chickens exposed to lipopolysaccharides from *Escherichia coli* and *Bacillus subtilis*

**DOI:** 10.14202/vetworld.2023.564-570

**Published:** 2023-03-22

**Authors:** Sandra Paola Rodríguez, Albeiro López Herrera, Jaime Eduardo Parra

**Affiliations:** Department of Animal Production, Faculty of Agricultural Sciences, Universidad Nacional de Colombia, Medellín campus 050034, Colombia

**Keywords:** antibiotic, enteritis, *Escherichia coli*, lipopolysaccharides

## Abstract

**Background and Aim:**

Intestinal infections are associated with Gram-negative bacteria like *Escherichia coli*. When eliminated by treatments during replication, *E. coli* release lipopolysaccharides (LPS) that can activate the intestinal immune system and increase the expression of cytokines, such as interleukin (IL)-8, IL-18, tumor necrosis factor-alpha (TNF-α), and interferon-gamma (IFN-γ), by the intestinal epithelium under pathological conditions. This study aimed to evaluate the addition of *Bacillus subtilis* to the duodenal gene expression of pro-inflammatory and anti-inflammatory cytokines in broilers exposed to LPS from *E. coli*.

**Materials and Methods:**

RNA was extracted using the Zymo Research total RNA commercial kit, according to the manufacturer’s specifications, from the intestinal tissue of the duodenum previously resuspended in the lysis buffer of the kit. The expression of the cytokines of interest was measured using the QuantiNova SYBR green real-time polymerase chain reaction kit (Qiagen). Transcript quantification was performed by the ΔΔC(t) method using glyceraldehyde 3-phosphate dehydrogenase as a normalizing constitutive gene.

**Results:**

For the measurement of pro-inflammatory (IL-8, IL-18, TNF-α, and IFN-γ) and anti-inflammatory (IL-10) cytokines, there was no statistically significant difference (p > 0.05) between the basal diet and the diet with antibiotic (avilamycin). There was a statistical difference (p < 0.05) between diets with LPS. The diet with *B. subtilis* presented the lowest expression; the results differed on each sampling day (days 14, 28, and 42).

**Conclusion:**

A decrease in the expression of pro-inflammatory cytokines (IL-8, IL-18, TNF-α, and IFN-γ) and an increase in IL-10 (anti-inflammatory) was observed; in this way, a balance of the inflammatory response to bacterial infection is achieved, suggesting that the use of *B. subtilis* as an additive in a broiler diet has a similar effect to that produced with antibiotic growth promoter.

## Introduction

Bacterial infections cause inflammatory processes that can cause alterations within the digestive system. One of the most efficient intestinal defense mechanisms is the production of pro-inflammatory cytokines [1, 2], such as interleukin (IL)-1 and IL-6, and chemokines, such as IL-8, interferon-gamma (IFN-γ), tumor necrosis factor-alpha (TNF-α), and IL-4 [3]. In this way, they collaborate with correct intestinal immunity against the microbial population [4].

The intestinal epithelium plays a fundamental role as a natural protective barrier of the organism. It protects from toxic substances in the intestinal lumen and from alterations caused by pathogens (*Escherichia coli*, *Salmonella* spp., and among others) that alter its permeability and facilitate the invasion of other microorganisms, affecting the ability to digest and absorb nutrients and, in general, the metabolism [5, 6]. Intestinal infections are characterized by increased Gram-negative bacteria, such as *E. coli*. When eliminated by treatments or death during replication, *E. coli* release lipopolysaccharides (LPS) [7] that can activate the intestinal immune system and increase the expression of cytokines, such as IL-8, IL-10, IL-18, and TNF-α, by the intestinal epithelium under pathological conditions [8]. Consequently, structural and functional alterations in the intestine are presented, such as an increase in the paracellular transport of molecules, diarrhea, and mortality [9]. Due to the prohibition of antibiotics as growth promoters in poultry in some countries, multifactorial diseases have emerged, causing enteritis and intestinal disorders of unknown origin, leading to negative impacts on health and productive performance [10]. Among them, intestinal dysbiosis, defined as the presence of qualitatively and/or quantitatively abnormal microbiota in the proximal parts of the small intestine, is associated with reduced digestibility of nutrients, impaired intestinal barrier function, bacterial translocation, and inflammatory response [11].

Cost-effective alternatives to antibiotics have been developed to reduce the increase in pathogen populations in broilers, including probiotics, prebiotics, symbiotics, exogenous enzymes, dietary/functional feeds, organic acids, phytochemicals, essential oils, and among others [12]. Spore-forming bacteria have an advantage when used as probiotics because spores remain protected from the environment before ingestion and inside the digestive tract [13].

Therefore, the study aimed to evaluate the addition of *Bacillus subtilis* to the duodenal gene expression of pro-inflammatory (IL-8, IL-18, TNF-α, and IFN-γ) and anti-inflammatory (IL-10) cytokines in boilers exposed to LPS from *E. coli*.

## Materials and Methods

### Ethical approval

All experimental procedures were carried out in accordance with the guidelines proposed by “The International Guiding Principles for Biomedical Research Involving Animals” [14]. This research was endorsed by “The Committee of Ethics in Animal Experimentation of the National University of Colombia,” Medellín campus (CEMED-013. May 04, 2016).

### Study period and location

The study was conducted from June to August, 2017. The fieldwork was carried out at the San Pablo Agrarian Station, belonging to the Universidad Nacional de Colombia, Medellín campus, located in the municipality of Rionegro, “El Tablacito” area, located at 2100 m above sea level, with a temperature between 12°C and 18°C, corresponding to a very humid zone in low Montano (bmh-MB).

### Animals

There were 144 1-day-old male chickens of the Avian Cobb500 line (Pronavicola, Colombia) housed in floor pens. The experimental period lasted 42 days. Chickens were raised according to the management procedures of a commercial farm.

### Experimental diets

Chickens were fed a commercial diet as a base (Table-1) without adding antibiotic, which was added in some antimicrobial treatments (AGPP; Avilamycin, Elanco, Medellín, Colombia). *B. subtilis* (Alterion^®^ Global Company, São Paulo^,^ Brazil) was used according to the manufacturer’s recommendation, 50 g/ton of feed to guarantee a dose of 10^8^ colony-forming units (CFU) and LPS from *E. coli* (LPS *E. coli*, serotype 0111:B4; Sigma-Aldrich, St. Louis, MO, USA) to induce intestinal inflammation *in vivo* [15], which are supplied directly in the feed as follows:

**Table-1:** Nutritional components of the basal diet designed in two stages for the study birds.

Nutritional contribution by stage		Initiation	Ending
	
Nutrients	Unit	Value	Value
Weight	kg	1.000	1.000
Humidity	%	10.926	10.845
EM birds	kcal/kg	3 152.165	3 299.259
Crude protein	%	21.474	19.976
Fat	%	8.301	10.213
Extract free of N	%	49.673	50.195
Crude fiber	%	2.927	2.801
Ashes	%	6.108	5.379
Calcium	%	0.997	0.832
Phosphorus available	%	0.418	0.360
Total phosphorus	%	0.648	0.580
Electrolyte balance	mEq/kg	216.164	191.553
Lysine	%	1.363	1.270
Methionine	%	0.650	0.602
Met+Cyst	%	0.993	0.924
Threonine	%	0.901	0.825
Tryptophan	%	0.242	0.222
Arginine	%	1.336	1.228
Isoleucine	%	0.881	0.815
Leucine	%	1.902	1.802
Valine	%	1.042	0.915
Histidine	%	0.547	0.509
Phenylalanine	%	1.061	0.989
Phenyl and tyrosine	%	1.953	1.828
Glycine	%	0.875	0.808
Alanine	%	1.158	1.103

- Basal diet 1 (D1): Commercial diet without antimicrobial addition, LPS, and *B. subtilis*
- Diet 2 (D2): D1 plus antimicrobial AGP (avilamycin, 10 ppm)
- Diet 3 (D3): D1 plus *B. subtilis* (50 ppm) to guarantee a daily consumption of 10^8^ CFU/animal and have adequate viability
- Diet 4 (D4): D1 plus 1.0 μg LPS/g feed (1 ppm)
- Diet 5 (D5): D1 plus 1.0 μg LPS/g feed (1 ppm) and antimicrobial AGP (avilamycin, 10 ppm)
- Diet 6 (D6): D1 plus 1.0 μg LPS/g feed (1 ppm) and *B. subtilis* at a rate of 50 ppm feed
- No information on production parameters, the sacrifice of chickens, sample collection procedures, days on which chickens were sacrificed, etc.

### RNA extraction and quantification of cytokine expression by real-time polymerase chain reaction (PCR)

RNA was extracted using the Zymo Research total RNA commercial kit, according to the manufacturer’s specifications, from the intestinal tissue of the duodenum previously resuspended in the lysis buffer of the kit. The expression of the cytokines of interest was measured using the QuantiNova SYBR Green RT-PCR Kit (Qiagen, Germantown, USA) in a LightCycler 480 real-time PCR system thermocycler (Roche Diagnostics, Indianapolis, USA). Briefly, 2.9 μL total RNA was amplified in a 10 μL reaction containing 5 μL of 2 Å QuantiNova SYBR Green RT-PCR master mix, 0.1 μL QuantiNova RT Mix, and 1 μL of each of sense and antisense primers (10 μM) [16].

The PCR tubes with 10 mL of the reaction mixture were incubated at 45°C for 10 min, followed by 95°C for 5 min and 40 cycles (95°C for 15 s, 60°C for 20 s, and 72°C for 30 s) and an extension period of 60°C for 60 s. The specificity of the reaction products was confirmed by melting temperature analysis. Transcript quantification was performed by the ΔΔC(t) method using glyceraldehyde 3-phosphate dehydrogenase as a normalizing constitutive gene [16]. Sequences of the used primers are presented in Table-2 [14–18].

**Table-2:** List of primers used for RNA amplification and expression quantification by real-time PCR of duodenal samples.

Primers	Sequence	Reference
IL-8	Forward CCGATGCCAGTGCATAGAGA Reverse GGTGTCTGCCTTGTCCAGAA	[14]
IL-18	Forward GCTGGAATGCGATGCCTTTT Reverse TCCACTGCCAGATTTCACCTC	[15]
TNF-α	Forward CCTGCTGGGGGAATGCTAGG Reverse AGCGTTGTCTGCTCTGTAGC	[16]
IL-10	Forward CAGACCAGCACCAGTCATCAG Reverse ATCCCGTTCTCATCCATCTTCTCG	[17]
IFN-γ	Forward CCAAGCTCCCGATGAACGAC Reverse TCCTCTGAGACTGGCTCCTTT	[14]
GAPDH	Forward CCAGCCGAGCCACATCGCTC Reverse AT GAGCCCCAGCCTTCTCCAT	[18]

PCR=Polymerase chain reaction, IL=Interleukin, TNF-α=Tumor necrosis factor-alpha, IFN-γ=Interferon-gamma, GAPDH=Glyceraldehyde-3-phosphate dehydrogenase

### Statistical analysis

The experiment was carried out using a completely randomized design in a split-plot arrangement. Each animal was assigned to one of six experimental diets. Each of the treatments (six diets) and three slaughter ages (days 14, 28, and 42) had a total of three repetitions. Statistical analysis was performed according to the PROC MIXED procedure of SAS version 9.3 (SAS Institute Inc., Cary, NC, USA, 2011). Differences between treatment means were determined by least squares and analysis of variance. To compare the means between each treatment, Duncan’s test was used to detect significance (p < 0.05) between the means.

## Results

**Figure-1:**
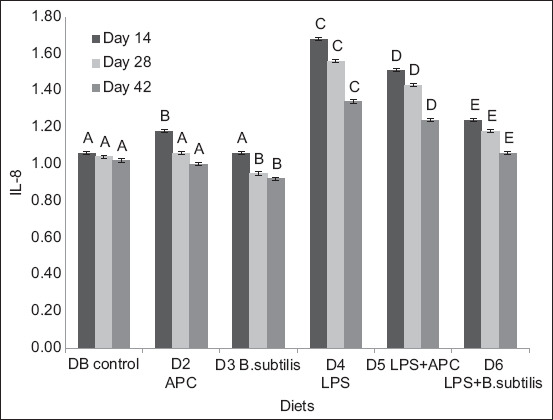
Expression of ARNm of interleukin-8 between each diet of the experiment and different sampling days. Basal diet (DB): Commercial diet without the addition of antimicrobial, lipopolysaccharides (LPS) and *Bacillus subtilis*. Diet 2 (D2): DB plus the addition of antimicrobial (avilamycin, 10 ppm). Diet 3 (D3): DB plus the addition of *B. subtilis* at a rate of 50 ppm of food. Diet 4 (D4): DB plus the addition of 1.0 μg of LPS/g in the feed. Diet 5 (D5): DB plus the addition of 1.0 μg of LPS/g in the feed and in the antimicrobial. Diet 6 (D6): DB plus the addition of 1.0 μg of LPS/g in the feed and *Bacillus subtilis* at a rate of 50 ppm of feed. ^A, B, C, D, E^ means with different superscripts are statistically different (p < 0.05). Means with a common superscript (variable under study) do not differ statistically (p > 0.05).

For IL-8, there was no significant statistical difference (p > 0.05) between DB and D2. There was a statistical difference during the 3 days of sampling between D3 and D4 and between D4 and D5 and D6, with D3 presenting the lowest expression of this pro-inflammatory cytokine, which translates into better results on sampling days (days 14, 28, and 42) (Figure-1).

**Figure-2:**
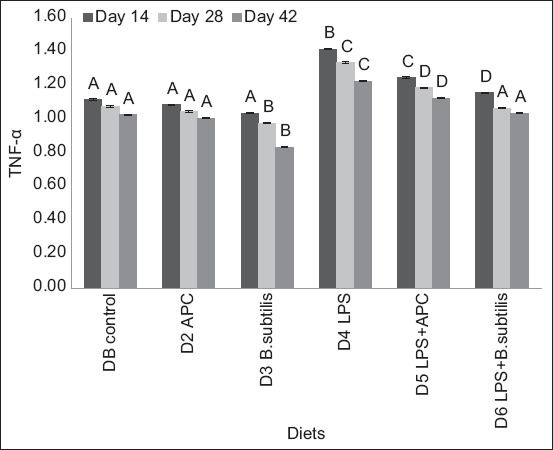
ARNm of tumor necrosis factor-alpha expression between each experiment diet and different sampling days. Basal diet (DB): Commercial diet without the addition of antimicrobial, lipopolysaccharides (LPS), and *Bacillus subtilis*. Diet 2 (D2): DB plus the addition of antimicrobial (avilamycin, 10 ppm). Diet 3 (D3): DB plus the addition of *B. subtilis* at a rate of 50 ppm of food. Diet 4 (D4): DB plus the addition of 1.0 μg of LPS/g in the feed. Diet 5 (D5): DB plus the addition of 1.0 μg of LPS/g in the feed and in the antimicrobial. Diet 6 (D6): DB plus the addition of 1.0 μg of LPS/g in the feed and *B. subtilis* at a rate of 50 ppm of feed. ^A, B, C, D, E^ means with different superscripts are statistically different (p < 0.05). Means with a common superscript (variable under study) do not differ statistically (p > 0.05).

To measure TNF-α, there was no significant statistical difference (p > 0.05) between D1 and D2. Compared to D2 (avilamycin), there was a statistical difference (p < 0.05) in diets with LPS (D4–D6). D3 (*B. subtilis*) presented a statistical difference (p < 0.05) in relation to diets with LPS. Results differed on each sampling day (days 14, 28, and 42; Figure-2).

**Figura-3:**
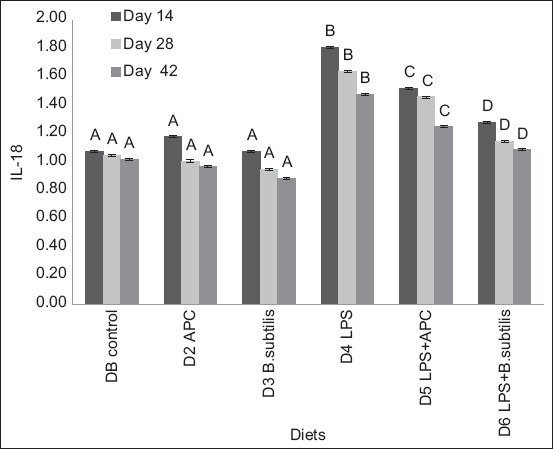
Expression of ARNm of interleukin-18 between each experiment diet and different sampling days. Basal diet (DB): Commercial diet without the addition of antimicrobial, lipopolysaccharides (LPS) and *Bacillus subtilis*. Diet 2 (D2): DB plus the addition of antimicrobial (avilamycin, 10ppm). Diet 3 (D3): DB plus the addition of *B. subtilis* at a rate of 50 ppm of food. Diet 4 (D4): DB plus the addition of 1.0 μg of LPS/g in the feed. Diet 5 (D5): DB plus the addition of 1.0 μg of LPS/g in the feed and in the antimicrobial. Diet 6 (D6): DB plus the addition of 1.0 μg of LPS/g in the feed and *B. subtilis* at a rate of 50 ppm of feed. ^A, B, C, D, E^ means with different superscripts are statistically different (p < 0.05). Means with a common superscript (variable under study) do not differ statistically (p > 0.05).

In IL-18 mRNA expression, there was no significant statistical difference (p > 0.05) between D1 and D2 in the 3 days of sampling. D2 (avilamycin) presented a statistical difference (p < 0.05) compared to diets with LPS and *B. subtilis* on days 14, 28, and 42. D3 (*B. subtilis*) presented the lowest expression, the best result during the experiment. It differed statistically with respect to diets with LPS (D4–D6) (Figure-3).

To measure TNF-α, there was no significant statistical difference (p > 0.05) between D1 and D2. Compared to D2 (avilamycin), there was a statistical difference (p < 0.05) with diets with LPS (D4–D6). D3 (*B. subtilis*) diet presented a statistical difference (p < 0.05) in relation to diets with LPS. Results differed on each sampling day (days 14, 28, and 42; Figure-2).

**Figure-4:**
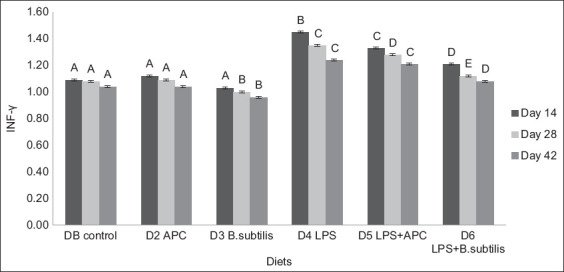
Expression of ARNm interferon-gamma between each experiment diet and different sampling days. Basal diet (DB): Commercial diet without the addition of antimicrobial, lipopolysaccharides (LPS), and *Bacillus subtilis*. Diet 2 (D2): DB plus the addition of antimicrobial (avilamycin, 10ppm). Diet 3 (D3): DB plus the addition of *B. subtilis* at a rate of 50 ppm of food. Diet 4 (D4): DB plus the addition of 1.0 μg of LPS/g in the feed. Diet 5 (D5): DB plus the addition of 1.0 μg of LPS/g in the feed and in the antimicrobial. Diet 6 (D6): DB plus the addition of 1.0 μg of LPS/g in the feed and *B. subtilis* at a rate of 50 ppm of feed. ^A, B, C, D, E^ means with different superscripts are statistically different (p < 0.05). Means with a common superscript (variable under study) do not differ statistically (p > 0.05).

When evaluating IFN-γ expression between D1 and D2, there was no significant statistical difference (p > 0.05) or between sampling days. For D2 and D3, there was a significant statistical difference (p < 0.05), with a greater presence on day 42. Between D3 and diets with LPS (D4–D6), there was a significant statistical difference (p < 0.05) on the different days of the study (Figure-4).

**Figure-5:**
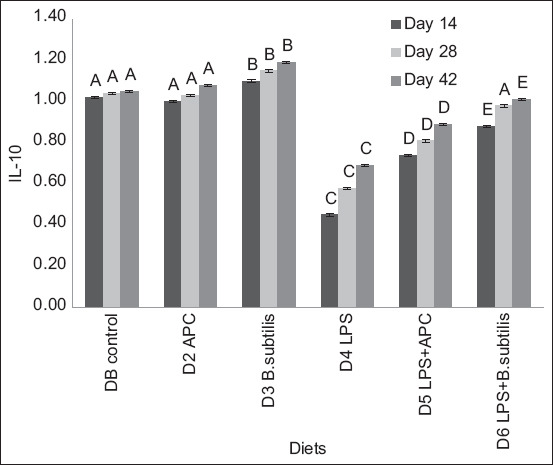
Expression of ARNm of interleukin-10 between each diet of the experiment and different sampling days. Basal diet (DB): Commercial diet without the addition of antimicrobial, lipopolysaccharides (LPS), and *Bacillus subtilis*. Diet 2 (D2): DB plus the addition of antimicrobial (avilamycin, 10 ppm). Diet 3 (D3): DB plus the addition of *B. subtilis* at a rate of 50 ppm of food. Diet 4 (D4): DB plus the addition of 1.0 μg of LPS/g in the feed. Diet 5 (D5): DB plus the addition of 1.0 μg of LPS/g in the feed and in the antimicrobial. Diet 6 (D6): DB plus the addition of 1.0 μg of LPS/g in the feed and *Bacillus subtilis* at a rate of 50 ppm of feed. ^A, B, C, D, E^ means with different superscripts are statistically different (p < 0.05). Means with a common superscript (variable under study) do not differ statistically (p > 0.05).

For IL-10 mRNA expression, there was no statistically significant difference (p > 0.05) between D1 and D2. Between D3 and D4, there was a significant statistical difference (p < 0.05), and the same occurred between D5 and D6. During sampling days 14 to 28 and 42, there was a significant statistical difference (p < 0.05) in each of the diets supplied (Figure-5).

## Discussion

In diets with *B. subtilis*, IL-8, IL-18, TNF-α, and IFN-γ expression decreased. Among chickens with dietary LPS addition, IL-8 and TNF-α expression was reduced in chickens fed diets supplemented with *B. subtilis* (D6). If an immune response occurs, cytokines or chemokines are released in amounts sufficient to suppress immune responses [19].

In diets containing *B. subtilis* (D3 and D6), the expression of pro-inflammatory cytokines (IL-8, IL-18, TNF-α, and IFN-γ) decreased compared to D1 and D2 (avilamycin). For pro-inflammatory cytokines, the lowest expression was in D3, containing the probiotic *B. subtilis*. When broilers were exposed to LPS, the expression of pro-inflammatory cytokines increased, with a decreased expression in D6 (LPS + *B. subtilis*). The probiotic protected the intestinal epithelium from the inflammatory process caused by LPS but did not completely recover it as in D3, showing regulation of the inflammatory response associated with monocyte mannose receptor and toll-like receptor 21 expression and activation of pattern recognition receptors, resulting in the production of cytokines that can link innate immunity with acquired immunity [20]. In contrast, the decrease in expression of pro-inflammatory cytokines as the age of the chicken increases and the increase in IL-10 suggested that *B. subtilis* has a role in maintaining immune homeostasis by preventing the triggering of a strong inflammatory response [19–21].

Lipopolysaccharides diets (D4–D6) had a higher IL-8, IL-18, TNF-α, and IFN-γ expression than those without LPS. These results were similar to those of [22], who reported that LPS injection increases the expression of pro-inflammatory cytokines, such as IL-1β and IL-6. In addition, the effect of inflammation on the growth rate and metabolic process could be mediated by cyclooxygenase-2 expression and the release of prostaglandins because they activate the neuronal pathways related to disease symptoms [23].

The presence of *E. coli* implies the suppression of cytokines, especially IL-10 but not TNF-α, which increases in the intestine of chickens fed with a diet with LPS [24]. Similar results were presented in this study with diets to which LPS from *E. coli* was added. Some studies showed that parental administration of the *Lactobacillus* probiotic isolate increases IL-18 and IL-12 expression [25]. In this study, in chickens fed with *B. subtilis*, IL-18 gene expression decreases but increases in diets with LPS from *E. coli*. This is necessary for regulating the physiological inflammation process responsible for the immune functioning of the normal intestinal mucosa and for maintaining the homeostatic balance between microbiota tolerance and reactivity to invasion by pathogens [26].

Decreased IL-18, STAT2, STAT4, MyD88, IFN-α, and IFN-γ expression is a response to the structural constituents of *Lactobacillus acidophilus*, similar to results in this study, where diets with *B. subtilis* (D3 and D6) presented a lower IL-18 expression. In this way, immune activities can be positively regulated before a pathogen challenge [27].

IL-8 is a cytokine that attracts and induces the accumulation of heterophils at the place of inflammation, an accumulation that usually causes tissue damage. In addition, it attracts peripheral blood monocytes and fibroblasts [28]. In this study, diets supplemented with avilamycin and *B. subtilis* decreased IL-8 expression mediated by LPS, compared to other diets, an important factor in attenuating the chemotactic capacity and activation of polymorphonuclear neutrophils and, in this way, regulating the inflammatory response. It is considered that *B. subtilis* has a modulating effect on IL-8 expression after exposure of chickens to LPS during the study time. Similar results were presented by Rocha-Ramírez *et al*. [25], who found a beneficial effect of two probiotic strains of *Bacillus* in modulating the induced inflammatory response *in vitro*.

Pro-inflammatory cytokines, such as TNF-α, IL-1β, and IL-6, released from monocytes and macrophages are decreased by including lactic acid bacteria and bifidobacteria [29]. Similar results were presented in this study in diets with supplementation of *B. subtilis* (D3) and avilamycin (D2). Tumor necrosis factor-alpha induces the endocytosis of binding proteins by increasing intestinal permeability [30]. *Bacillus subtilis* exerts an immunomodulatory function by suppressing the gene expression of pro-inflammatory cytokines, as it contains surfactin that induces protective effects on the intestinal mucosa [31].

Interferon-gamma in poultry is an essential cytokine in host defense against pathogens, as demonstrated in various infection models [32]. However, this IL decreases its expression as time passes, as evidenced in this study at 28 and 42 days. According to Saki *et al*. [33], this can be explained by the fact that probiotics (in this study, *B. subtilis*) can regulate inflammatory-type responses due to their stabilizing effect on the intestine by stimulating the balanced secretion of anti-inflammatory and pro-inflammatory cytokines, thus preventing the growth of pathogens that can cause diseases in chickens [34, 35].

Interleukin-10 is an immunoregulatory cytokine that plays an important inhibitory role in various inflammatory responses and controls the host immune response to limit cell damage during inflammation by inhibiting pro-inflammatory cytokines, such as IL-12, IFN-γ, TNF-α, IL-1β, IL-2, and IL-6 [36]. The increase in IL-10 values observed in diets supplemented with *B. subtilis* (D3 and D6) suggests stimulation of anti-inflammatory factors. This trend supported the works of [37, 38], although the detailed mechanism through which *B. subtilis* modifies IL-10 expression is not well understood yet [39].

## Conclusion

*Bacillus subtilis* has a beneficial effect similar to that of APC as a modulator of the inflammatory response, decreasing the expression of pro-inflammatory cytokines (IL-8, IL-18, TNF-α, and IFN-γ) and increasing that of IL-10 (an anti-inflammatory cytokine), so that it maintains a balance of the inflammatory response in bacterial infection, relevantly in chickens exposed to LPS. Therefore, *B. subtilis* could be used as a replacement for APC in broiler diets.

## Authors’ Contributions

SR and JP: Designed the study and drafted the manuscript. SR, JP and AL: Methodology, data collection, formal analysis, and technical help during the experiments. All authors have read, reviewed, and approved the final manuscript.

## Data Availability

The supplementary data can be obtained from the corresponding author upon request.
